# Rhino-Orbital-Cerebral Mucormycosis With Intracranial Extension in a Patient With Diabetic Ketoacidosis

**DOI:** 10.7759/cureus.114573

**Published:** 2026-08-15

**Authors:** Norlando José Chávez Durón, Lorenzo E Aragón Conrado, Francgiliz J Robleto, Ledixa Zeledon, María Gabriela Zelaya Aróztegui, Leyda O Alonso Chavarría, María Cooper

**Affiliations:** 1 Internal Medicine, Hospital Escuela Óscar Danilo Rosales Argüello (HEODRA), León, NIC; 2 Medical Education, El Hospital Militar Escuela “Dr. Alejandro Dávila Bolaños”, Managua, NIC; 3 Medical Education, Escuela de Medicina Teniente Coronel y Doctor Sergio Martinez Ordoñez, Managua, NIC; 4 Internal Medicine, El Hospital Militar Escuela “Dr. Alejandro Dávila Bolaños", Managua, NIC; 5 General Medicine, National Autonomous University of Nicaragua, León, NIC; 6 Internal Medicine, National Autonomous University of Nicaragua, León, NIC; 7 Pediatric Medicine, National Autonomous University of Nicaragua, León, NIC; 8 Gastroenterology, NewYork–Presbyterian Hospital, New York, USA

**Keywords:** amphotericin b, intracranial extension, orbital cellulitis, rhino-orbital-cerebral mucormycosis, rocm, severe diabetic ketoacidosis

## Abstract

Rhino-orbital-cerebral mucormycosis (ROCM) is a rare but highly aggressive fungal infection that predominantly affects immunocompromised individuals, with diabetic ketoacidosis (DKA) being one of the most important predisposing factors. We report the case of a 44-year-old woman from León, Nicaragua, with poorly controlled type 2 diabetes mellitus who presented with fever, upper respiratory symptoms, palatal ulceration, and progressive orbital involvement. She was admitted in severe DKA and diagnosed with invasive ROCM based on histopathological findings of broad, ribbon-like, aseptate hyphae with right-angle branching. Despite early initiation of amphotericin B and subsequent surgical debridement, the infection proved fatal. This case highlights the need for timely recognition, combined antifungal and surgical management, and optimization of underlying metabolic conditions to improve outcomes in ROCM.

## Introduction

Rhino-orbital-cerebral mucormycosis (ROCM) is a rare but highly aggressive fungal infection that primarily affects immunocompromised individuals. Uncontrolled type 2 diabetes mellitus - especially when complicated by diabetic ketoacidosis (DKA) - is one of the strongest predisposing factors. In this metabolic state, impaired neutrophil function and increased free serum iron favor rapid fungal proliferation, most commonly by Rhizopus species [1].

The infection typically begins as necrotizing sinusitis and may rapidly extend into the orbit and central nervous system. Clinical manifestations include unilateral facial pain, fever, sinusitis, ophthalmoplegia, or vision loss, with progression to neurological deficits due to vascular invasion [2]. Given its fulminant course and high lethality, timely diagnosis and prompt initiation of antifungal and surgical management are essential, particularly in patients with uncontrolled diabetes and DKA. Here, we report a fatal case of rhino-orbital-cerebral mucormycosis (ROCM) in a 44-year-old woman with poorly controlled type 2 diabetes mellitus presenting with diabetic ketoacidosis. Despite early initiation of antifungal therapy, the patient experienced rapid disease progression with orbital and intracranial involvement, requiring extensive surgical debridement and right eye enucleation before ultimately succumbing to the infection. This case highlights the aggressive clinical course of ROCM and emphasizes the importance of early recognition, prompt multidisciplinary management, and timely surgical intervention, particularly in resource-limited settings where delays in diagnosis and treatment may adversely affect outcomes.

## Case presentation

A 44-year-old woman from rural León, Nicaragua, presented with 14 days of fever and upper respiratory symptoms. She was illiterate, a housewife, and married. Medical history included poorly controlled type 2 diabetes mellitus, untreated for three months.

On admission, the patient's vital signs and initial laboratory findings were consistent with severe DKA (Table 1).

**Table 1:** Initial vital signs and laboratory findings on admission MCV, mean corpuscular volume; BP, blood pressure; HR, heart rate; RR, respiratory rate; eGFR, estimated glomerular filtration rate.

Parameter	Result	Reference range
White blood cell count	18.6 ×10³/µL	4.0–10.0 ×10³/µL
Neutrophils	88%	40–75%
Hemoglobin	9.8 g/dL	12.0–16.0 g/dL
MCV	72 fL	80–100 fL
Blood glucose	540 mg/dL	70–99 mg/dL
Potassium	3.2 mmol/L	3.5–5.0 mmol/L
Chloride	112 mmol/L	98–107 mmol/L
Lactate	3.8 mmol/L	0.5–2.0 mmol/L
Creatinine	1.52 mg/dL	0.50–1.10 mg/dL
eGFR	43 mL/min/1.73 m²	≥90 mL/min/1.73 m²
Serum osmolality	350 mOsm/kg	275–295 mOsm/kg
Urine glucose	3+ (positive)	Negative
Urine ketones	3+ (positive)	Negative

Physical exam showed mucocutaneous pallor, exophytic oral lesions, and a 2-3 cm ulcer on the right hard palate with necrotic base and irregular borders. There was purulent discharge from the right auditory canal, right palpebral ptosis with ophthalmoplegia, proptosis, and periorbital and hemifacial edema. Other findings included a deviated septum and hyperpigmented skin lesions with perifocal hyperkeratosis and edema.

She was admitted to ICU with severe DKA (Kussmaul respiration, fruity odor, epigastric tenderness). Initial laboratory evaluation demonstrated leukocytosis with neutrophilia, grade II microcytic anemia, severe hyperglycemia, metabolic acidosis with hyperlactatemia, mild hypokalemia, hyperchloremia, acute kidney injury, glucosuria, and ketonuria (Table 1).

**Figure 1:**
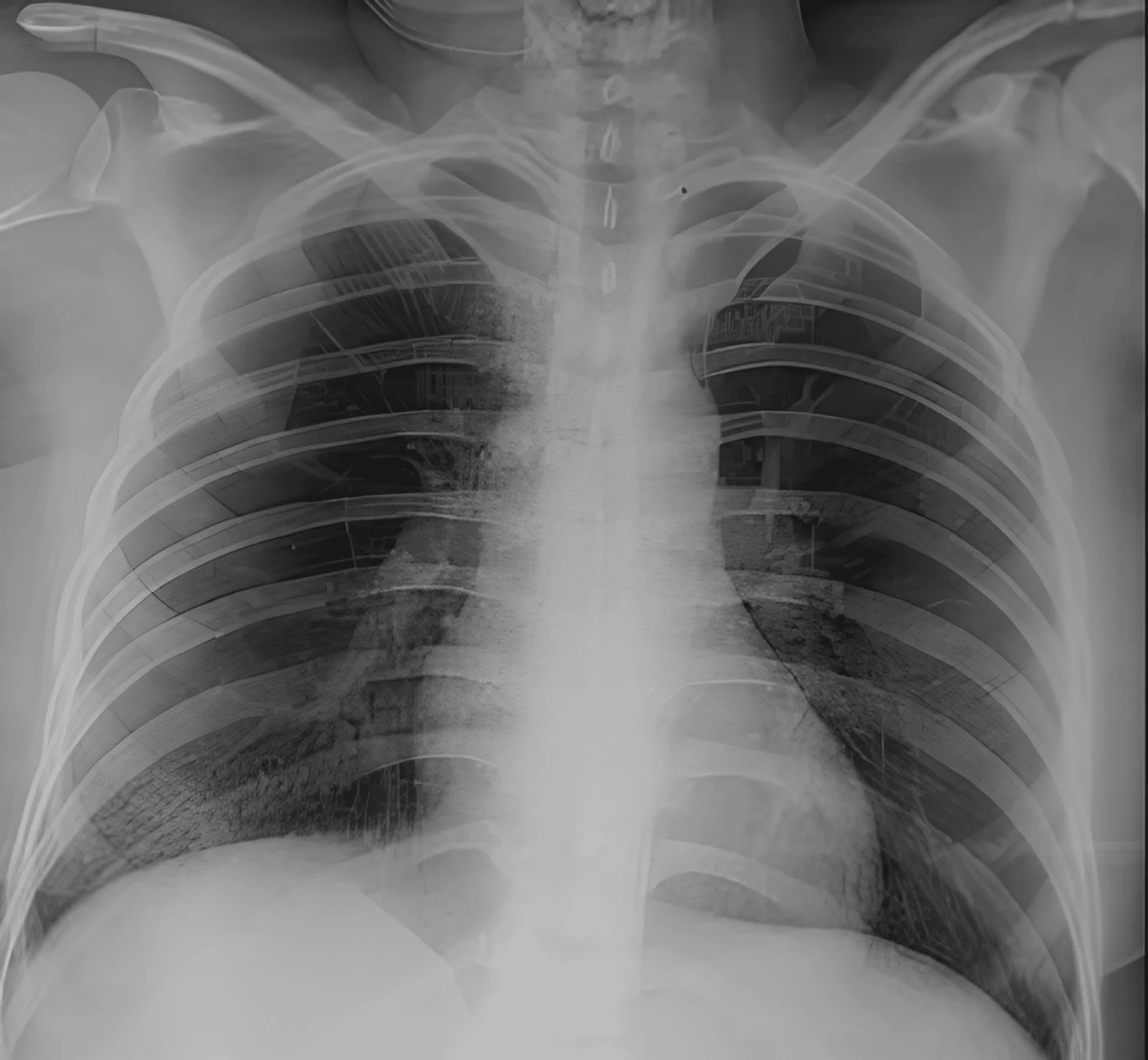
Normal Chest X-ray Chest radiograph revealed no focal pulmonary infiltrates, pleural effusion, or other acute cardiopulmonary abnormalities

Chest X-ray showed no infiltrates or effusion (Figure 1).

**Figure 2:**
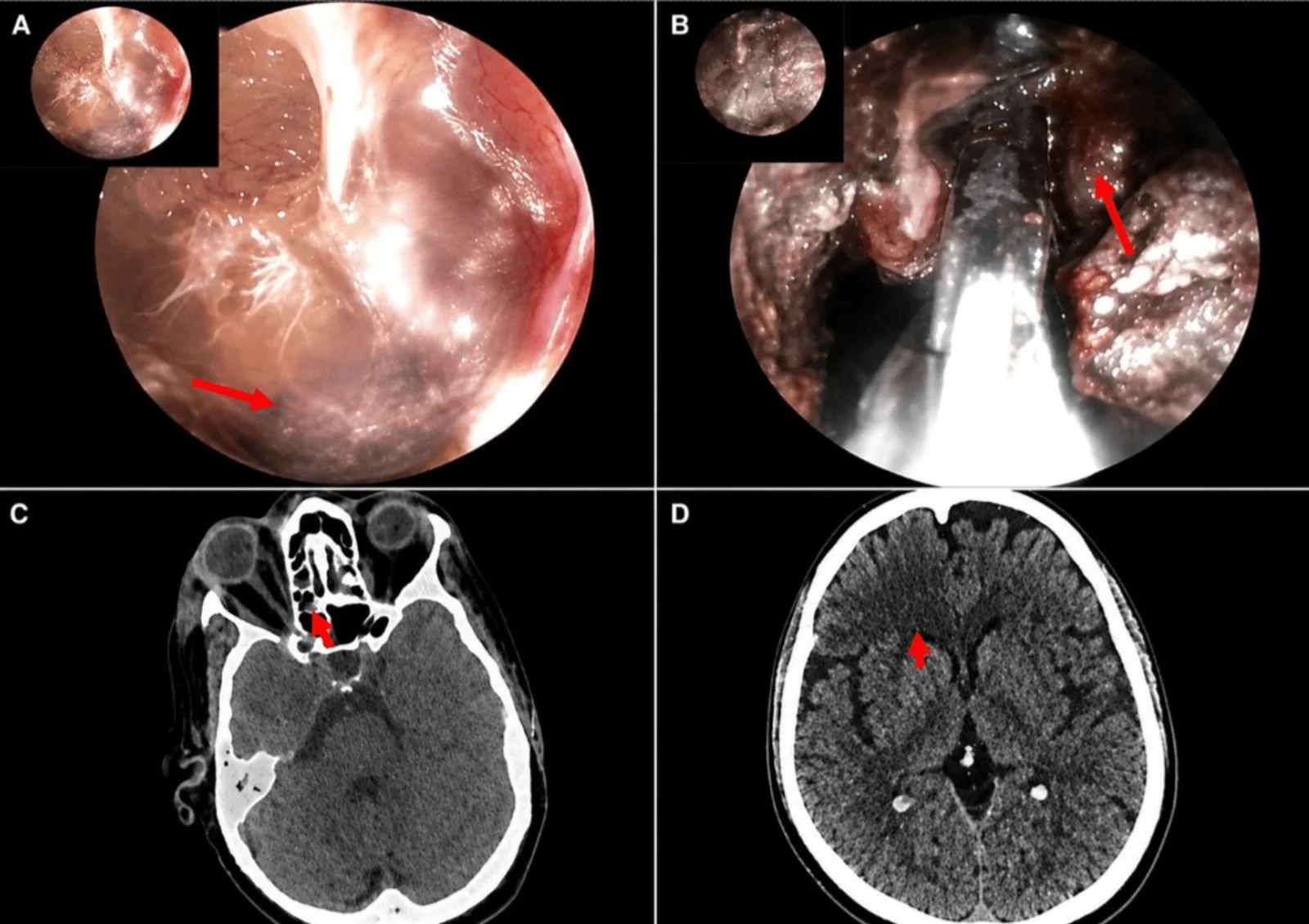
Endoscopic and CT findings in rhino-orbito-cerebral fungal Infection (A) Nasal endoscopy demonstrating necrotic debris involving the right nasal turbinate (arrowhead); (B) Endoscopic debridement of necrotic tissue from the right nasal cavity (arrowhead); (C) Axial computed tomography of the paranasasal sinuses showing opacification of the ethmoid and sphenoid sinuses with extension into the right orbit (arrowhead); (D) Axial non-contrast brain computed tomography demonstrating an irregular hypodense area in the right frontal lobe, suggestive of cerebral edema (arrowhead).

Head and paranasal sinus CT showed grade II right proptosis, orbital fat plane loss, mucosal thickening in ethmoid, sphenoid, and maxillary sinuses with hyperdense content, and right frontal cerebral edema suggesting intracranial extension (Figure 2).

Nasal endoscopy revealed necrotic debris in the right turbinate (Figure 2).

**Figure 3:**
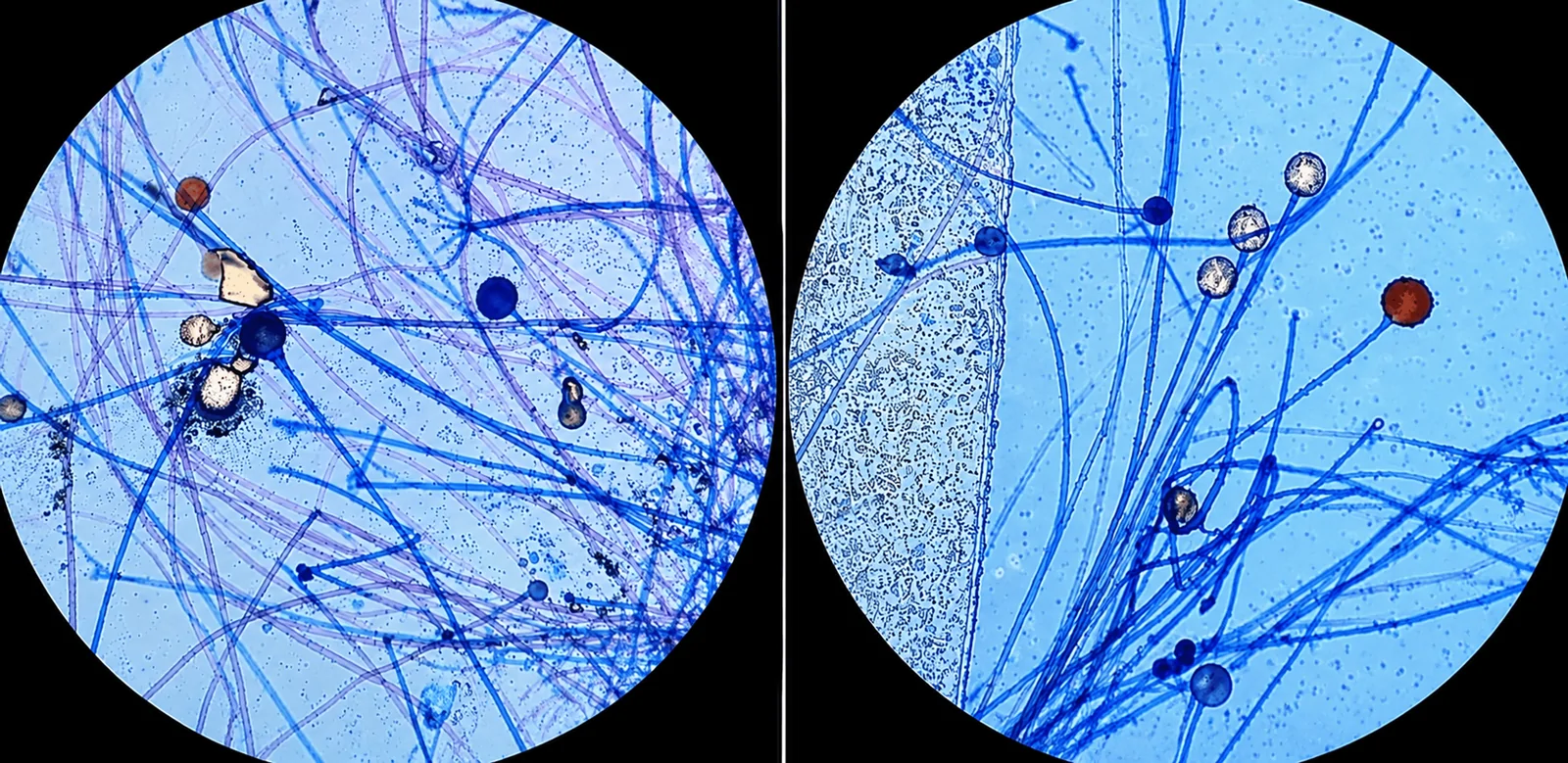
Lactophenol cotton blue microscopy of Mucorales Representative microscopic fields stained with lactophenol cotton blue demonstrating broad, ribbon-like, aseptate hyphae with right-angle branching and spherical sporangia, characteristic of fungi belonging to the order Mucorales.

Lactophenol blue preparation of ethmoidal tissue demonstrated broad, aseptate hyphae with 90° branching, as well as spherical saccular fructification structures (sporangia) containing yellow-brown pigmented material (Figure 3).

Ear canal culture grew Staphylococcus aureus colonization; blood and urine cultures were negative.

Initial management consisted of antifungal therapy with amphotericin B deoxycholate of 1 mg/kg/day. Three days later, right eye enucleation combined with extensive surgical debridement of necrotic tissue was performed. The patient received amphotericin B for two weeks, during which serial surgical debridement was performed every 48 hours. Despite treatment, the patient developed recurrent seizures and progressive neurological deterioration, ultimately leading to cardiorespiratory collapse. The infection proved fatal.

## Discussion

First described in 1876 by Fürbinger, mucormycosis is caused by fungi of the order Mucorales, which are widely distributed in the environment [1,2]. ROCM is the most common clinical form, accounting for approximately 67.7% of cases [3]. It is strongly associated with diabetes mellitus (54-76%), particularly when complicated by DKA, in which hyperglycemia impairs neutrophil function and acidosis increases free serum iron, promoting rapid fungal growth [4].

Early symptoms are often nonspecific, including unilateral facial pain, fever, retro-orbital headache, hyposmia, and nasal congestion. These may progress to black nasal discharge or necrotic eschars on the palate or face. Orbital and CNS involvement - manifested by ophthalmoplegia, proptosis, altered mental status, or focal neurological deficits - indicates advanced disease and is associated with a poor prognosis [1].

Timely diagnosis is critical. Although polymerase chain reaction (PCR)-based assays for fungal DNA continue to evolve, their sensitivity remains suboptimal, with false negatives reported in up to 50% of cases [3,5]. Histopathological confirmation through tissue biopsy is therefore considered the gold standard. In this case, microscopy revealed broad, aseptate, ribbon-like hyphae with right-angle branching, characteristic of Mucorales species, supporting the diagnosis.

Management of ROCM requires an urgent, combined approach that includes systemic antifungal therapy, aggressive surgical debridement, and correction of underlying metabolic disturbances. Liposomal amphotericin B at 5-10 mg/kg/day is the preferred treatment due to its improved safety profile, although amphotericin B deoxycholate remains the only feasible option in some resource-limited settings [6]. Surgical intervention is essential, as the angioinvasive nature of the fungus leads to extensive tissue necrosis with poor antifungal penetration [7]. Multiple debridements are often required to achieve adequate source control [6].

In this patient, amphotericin therapy was initiated promptly with intensive intravenous hydration to maintain a positive fluid balance; however, surgical debridement occurred three days after admission due to the absence of a dedicated mucormycosis protocol at our hospital and delays in the surgical team's decision to proceed, during which time the infection progressed with intracranial extension. Combined with the severity of her DKA and persistent metabolic instability, these factors likely contributed to the fatal outcome. This case illustrates that uncontrolled diabetes remains a major modifiable risk factor associated with ROCM-related mortality and highlights the importance of early, coordinated medical and surgical intervention-particularly in low-resource settings where diagnostic and therapeutic delays are more likely.

## Conclusions

ROCM is a rapidly progressive and frequently fatal opportunistic infection that predominantly affects patients with uncontrolled diabetes mellitus, particularly in the setting of DKA. This case highlights the aggressive angioinvasive nature of the disease and the devastating consequences of delayed diagnosis and surgical intervention. Early recognition of warning signs, including orbital involvement and necrotic sinonasal or palatal lesions, should prompt immediate multidisciplinary management with prompt initiation of antifungal therapy, aggressive surgical debridement, and metabolic stabilization. In resource-limited settings, where access to liposomal amphotericin B and standardized treatment protocols may be restricted, heightened clinical suspicion and expedited coordinated care are essential to improve outcomes and reduce mortality.
